# Aquaporins in ovine amnion: responses to altered amniotic fluid volumes and intramembranous absorption rates

**DOI:** 10.14814/phy2.12868

**Published:** 2016-07-20

**Authors:** Cecilia Y. Cheung, Debra F. Anderson, Robert A. Brace

**Affiliations:** ^1^ Department of Obstetrics and GynecologyOregon Health and Science UniversityPortlandOregon; ^2^ Center for Developmental HealthOregon Health and Science UniversityPortlandOregon

**Keywords:** Aquaporin, intramembranous absorption, oligohydramnios, polyhydramnios

## Abstract

Aquaporins (AQPs) are transmembrane channel proteins that facilitate rapid water movement across cell membranes. In amniotic membrane, the AQP‐facilitated transfer of water across amnion cells has been proposed as a mechanism for amniotic fluid volume (AFV) regulation. To investigate whether AQPs modulate AFV by altering intramembranous absorption (IMA) rate, we tested the hypothesis that AQP gene expression in the amnion is positively correlated with IMA rate during experimental conditions when IMA rate and AFV are modified over a wide range. The relative abundances of AQP1, AQP3, AQP8, AQP9, and AQP11 mRNA and protein were determined in the amnion of 16 late‐gestation ovine fetuses subjected to 2 days of control conditions, urine drainage, urine replacement, or intraamniotic fluid infusion. AQP mRNA levels were determined by RT‐qPCR and proteins by western immunoblot. Under control conditions, mRNA levels among the five AQPs differed more than 20‐fold. During experimental treatments, mean IMA rate in the experimental groups ranged from 100 ± 120 mL/day to 1370 ± 270 mL/day. The mRNA levels of the five AQPs did not change from control and were not correlated with IMA rates. The protein levels of AQP1 were positively correlated with IMA rates (*r* ^2^ = 38%, *P* = 0.01) while the remaining four AQPs were not. These findings demonstrate that five AQPs are differentially expressed in ovine amnion. Our study supports the hypothesis that AQP1 may play a positive role in regulating the rate of fluid transfer across the amnion, thereby participating in the dynamic regulation of AFV.

## Introduction

Early studies showed that infusion of warm water into the amniotic fluid of pregnant sheep resulted in rapid absorption of water into the fetal circulation that was independent of fetal swallowing (Gilbert and Brace 1989, 1990). The rapid absorption is mediated by the transfer of water across the amnion into the underlying fetal vasculature with the amnion as the rate‐limiting layer, and this process is referred to as intramembranous absorption (IMA). More recent studies have determined that IMA rate not only modulates but is also the primary regulator of amniotic fluid volume (AFV) under a variety of experimental conditions (Daneshmand et al. 2003; Thurlow and Brace 2003; Brace et al. 2004, 2014a,b; Gesteland et al. 2009; Robertson et al. 2009; Anderson et al. 2013).

Subsequent to the observation of water transfer across the intramembranous pathway, aquaporin (AQP) proteins were recognized as water channels that facilitate the movement of water across cell membranes (Agr et al. 1993). In human amnion, the transcripts and proteins of five AQPs (AQP1, AQP3, AQP8, AQP9, and AQP11) of the 13 known mammalian AQPs were present, while the remaining eight AQPs were not detected (Prat et al. 2012; Bednar et al. 2015). In dog amnion, AQP1, AQP3, AQP5, AQP8, and AQP9 were identified (Aralla et al. 2007). In fetal sheep, the most frequently used model for studying the regulation of AFV, AQP expression in the amnion has been explored only in a few studies. AQP3 mRNA was found to localize in trophoblast cells of the chorion and fibroblasts of the amnion, while AQP1 was absent from amniotic epithelial cells but present in vascular endothelial cells of amnion and chorion (Johnston et al. 2000). AQP9 was found to express in ovine amniotic epithelium (Wang et al. 2005). The presence of AQP8 and AQP11 in amnion of sheep has not been reported.

The observation of AQP expression in the amnion led to the speculation that AQPs may be involved in facilitating amnion water transport through the intramembranous pathway, and thus may play an important role in regulating AFV (Liu and Wintour 2005; Beall et al. 2007a,b; Liu et al. 2008). However, their participation in AFV regulation has not been explored. Current concepts are that the IMA of water across the amnion consists of two components: a minor component of bidirectional passive transfer primarily through AQP water channels and a major component of outwardly directed bulk vesicular transport of amniotic water and solutes (Brace et al. 2004, 2014b; Adams et al. 2005; Cheung and Brace 2008; Gesteland et al. 2009; Anderson et al. 2013). Our recent studies showed that the vesicular component of IMA is regulated by a renally derived stimulator and a nonrenal, nonpulmonary inhibitor that act in concert to modulate the unidirectional vesicular transport process (Anderson et al. 2013; Brace et al. 2014a,b). However, whether there were concomitant changes in AQP water channel activities when IMA rates were modified remain unknown.

Changes in passive transfer of water across the amnion induced by experimental manipulations may be difficult to detect because passive water flux apparently represents a small fraction of total IMA (Brace et al. 2014b). Therefore, in order to specifically address the question of whether the AQP water channels play a dynamic role in regulating AFV, we designed this study to investigate the expression profiles of AQP1, AQP3, AQP8, AQP9, and AQP11 mRNA and protein in ovine amnion and determine their responses to experimental conditions of decreased or increased IMA rate and AFV. We hypothesized that the mRNA and protein levels of the five AQPs in the amnion would be positively correlated with IMA rate and would be altered in response to changes in IMA rate, consistent with their potential role in modulating IMA and thus AFV.

## Materials and Methods

### Animal studies

These studies were approved by our Institutional Animal Care and Use Committee (IACUC) and were conducted in accordance with the National Research Council's *Guide for the Care and Use of Laboratory Animals*.

Sixteen late‐gestation pregnant ewes with singleton fetuses were used for the study. The surgical preparation and maintenance have been described in detail (Zhu et al. 2010; Anderson et al. 2013). Briefly, under inhalation anesthesia, the fetuses were fitted with catheters in the vasculature, amniotic fluid, trachea, and urinary bladder as well as a flow probe around the cervical esophagus. The ewes received prophylactic analgesics and the fetuses received prophylactic antibiotics administered into the amniotic fluid at surgery and for 2 days following surgery.

Experiments began 5 days after surgery [at 126.4 ± 0.5 (SE) days gestation, *n* = 16] and continued over a 2‐day period. The 2‐day period was chosen as our previous studies suggest that AFV normally reaches a steady state 1–2 days after a volume or flow disturbance (Anderson et al. 2013; Brace et al. 2014a,b). During the experiments, urine flow rate, lung liquid secretion rate, and swallowed volume were continuously monitored. AFV was determined at the beginning and end of each experiment, and the average IMA rate during the 2‐day experimental period was calculated from the change in AFV plus the time‐integrated flow rates (Zhu et al. 2010; Anderson et al. 2013; Brace et al. 2014a). Four fetuses were studied in each of the four groups: (1) control, (2) continuous fetal urine drainage without replacement, (3) urine drainage with isovolumic replacement (lactated Ringer's solution), and (4) continuous intraamniotic fluid infusion (lactated Ringer's solution at 2 L/day, 1.39 mL/min). These experimental conditions were chosen in order to vary IMA rate independent of AFV: urine drainage reduces both IMA rate and AFV; urine replacement reduces IMA rate while AFV increases; and intraamniotic infusion increases both IMA rate and AFV (Anderson et al. 2013; Brace et al. 2014a). On day 2 at completion of the protocols, the animals were euthanized with an IACUC approved intravenous euthanasia solution. Samples of amnion were collected immediately and either placed in RNA*later* ^*®*^ or in liquid nitrogen prior to storage at −80°C.

### Aquaporin real‐time RT‐qPCR

Amnion tissues were homogenized in a bead‐mill TissueLyser (MM301, Retsch GMBH & Co., Haan, Germany) and RNA was extracted using an RNeasy Kit (Qiagen, Inc., Valencia, CA). Single‐strand cDNA synthesis was carried out by reverse transcription using MultiScribe reverse transcriptase and random primers in the presence of RNase inhibitor (Applied Biosystems, Life Technologies, Foster City, CA). Sample cDNA (25 ng/sample) was amplified with ovine‐specific primers and probes for ovine AQP1, AQP3, AQP8, AQP9, or AQP11 (Table 1) custom designed using Primer Express^®^ Software v3.0 (Applied Biosystems, Thermo Fisher Scientific, Foster City, CA). The amplified sequences were validated by sequencing and alignment to the consensus ovine sequences. For AQP8 and AQP11 in which published full‐length ovine sequences were not available in the GenBank database, the primer sequences were designed based on the predicted *Ovis aries* nucleotide sequences (GenBank) and amplified sequences were aligned to the respective *Bos taurus* sequences published in Genbank. For the amplification reaction, *TaqMan* Gene Expression Assays (Applied Biosystems) were used in a ViiA7 Real‐Time PCR System (Applied Biosystems) with a temperature profile of an initial two‐step hold at 50°C for 2 min and 95°C for 10 min, followed by 40 cycles of 15 sec at 95°C and 1 min at 60°C. Two endogenous references, 18S ribosomal RNA and ovine RPLP0 mRNA, were used as house‐keeping genes as we have documented that their expression levels in various ovine tissues were unchanged under diverse experimental conditions. In each AQP PCR reaction, samples were analyzed in triplicates. A standard curve was incorporated for each of the endogenous references as well as for each AQP gene. The PCR amplification efficiency for each target was calculated from the respective standard curves. The average PCR efficiencies were as follows: AQP1 91.5 ± 5.5%, AQP3 93.5 ± 0.2%, AQP8 91.2 ± 1.8%, AQP9 82.4 ± 1.0%, AQP11 83.3 ± 0.2%, 18S 91.8 ± 2.0%, and RPLP0 88.0 ± 0.6%. This allowed for normalization of C_T_ values to 100% amplification efficiency for each endogenous reference and each AQP needed for comparative analysis.

**Table 1:** Custom‐designed ovine‐specific aquaporin primers and probes

Target gene	Primer and probe nucleotide sequence
AQP1	
Forward	TGCTCAGCTGCCAGATCAGTA
Reverse	CCACGCACTGGGCAATG
Probe	5′‐6FAMTCCGGGCCATCATGTAMGBNFQ‐3′
AQP3	
Forward	ACCCTTCTGGACACTTGGACAT
Reverse	GCCGTGCCGATGAACTG
Probe	5′‐6FAMTCAATGGCTTCTTCGMGBNFQ‐3′
AQP8	
Forward	CCGAGGAGAGGTTCTGGAATG
Reverse	CCGACTGCTGGACTGTCA
Probe	5′‐6FAMAAAGGCCGCCCCGGTCGMGBNFQ‐3′
AQP9	
Forward	GGAGTCCCCAGAGGCCTAGA
Reverse	AGGAGGCGATGGTGACAATC
Probe	5′‐6FAMCTGTTGTCATTGGCTTCMGBNFQ‐3′
AQP11	
Forward	GCCCAAAGCGGTCATCATA
Reverse	AGCAAAGCGCTGTGGAAAAT
Probe	5′‐6FAMAGGCTGTCTGCTCTT MGBNFQ‐3′

To determine differences in relative transcript quantities among AQPs, the fold change of individual AQP mRNA relative to AQP9 mRNA in the control group was calculated using the comparative C_T_ method (Schmittgen and Livak 2008). AQP9 in the control group was used as the calibrator because it was the least expressed target. Each efficiency‐corrected triplicate mean AQP C_T_ value was normalized to the geometric mean of the C_T_ values (∆C_T_) for the two respective house‐keeping genes. The fold change of individual AQP mRNA was calculated using the formula: 2^−∆∆C^_T_ (Schmittgen and Livak 2008), where ∆C_T_ denotes the target AQP C_T_ value normalized to the house‐keeping genes and ∆∆C_T_, the difference from ∆C_T_ of control AQP9. Such calculations allowed comparisons of relative mRNA quantities among the five AQPs and between the experimental groups.

### Western immunoblot for aquaporin proteins

Frozen amnion tissues were homogenized and membrane proteins were extracted using a Native Membrane Protein Extraction Kit (Calbiochem, EMD Millipore, Billerica, MA). The procedure selectively yielded integral membrane proteins and membrane‐associated proteins separate from cytosolic proteins. Membrane protein fractions (30 *μ*g/sample) were subjected to SDS‐PAGE (sodium dodecyl sulfate‐polyacrylamide gel electrophoresis) in precast 12% polyacrylamide gels (Bio‐Rad Laboratories, Hercules, CA) and electro‐transferred onto Immobilon‐P PVDF membranes (0.45 *μ*m, EMD Millipore). Each gel consisted of 16 samples (Four animals from each of the four groups) and each group of 16 samples was analyzed on separate gels. The membranes were blocked with 5% (w/v) nonfat dry milk in TBS‐Tween 20 (pH 7.6) and incubated with the primary antibody at 4°C overnight. For detection of ovine AQP proteins, specific anti‐human antibodies (Santa Cruz Biotechnology, Inc. Santa Cruz, CA) for AQP1, AQP3, AQP8, AQP9, and AQP11 were used at dilutions indicated in Table 2. These primary antibodies have a broad range of species specificity suitable for western immunoblotting of proteins from multiple species including those of human, rat, mouse, bovine, and porcine origin. In the ovine amnion membrane fraction, each of the five AQP antibodies detected a discrete protein band at the molecular weight specifically recognized by the antibody used: AQP1, 35 kD glycosylated form; AQP3, 36 kD; AQP8, 34 kD; AQP9, 33 kD; and AQP11, 36 kD (consistent with manufacturer published molecular weights detected by the respective AQP antibodies). To confirm the specificity of the primary antibodies, we performed positive control analyses using proteins from ovine amnion, chorion, placental cotyledon, and allantois. Positive signals were obtained for each of the five antibodies in all tissues tested. Signal intensity for AQP1 was highest in amnion and least in allantois, while AQP3, AQP9, and AQP11 were most expressed in chorion and cotyledon but least in amnion. These observations are consistent with the previous reports (Johnston et al. 2000; Wang et al. 2005). Endogenous *β*‐actin was used as an internal reference. After AQP antibody treatment, the PVDF membranes were stripped by incubating in the antibody stripping buffer (ReBlot Plus, EMD Millipore, Billerica, MA) for 20 minu at room temperature to dissociate the primary antibodies and reprobed with an anti‐human *β*‐actin antibody (Table 2). Following antibody treatment, the membranes were incubated with horseradish peroxidase‐conjugated secondary antibodies (Table 2) for 45 minutes at room temperature. Protein expression was detected using enhanced chemiluminescent substrate (Pierce^®^ ECL2 Western Blotting Substrate, Thermo Scientific) and exposure to autoradiography using BlueUltra autoradiography film (GeneMate, BioExpress, Kaysville, UT). The autoradiograms were scanned, images captured, and intensity of the signal for the specific protein was determined by densitometry using Image Studio Lite software (Version 3.1, Li‐Cor Bioscience, Lincoln, NE). For comparison among experimental groups, the mean protein quantity under control conditions for each of the five AQPs was used as the calibrator for the respective AQP. Comparisons of protein levels among the five AQPs in individual groups were not analyzed as the setup format of the western blot would not allow cross comparisons among individual gel blots.

**Table 2:** Human‐specific aquaporin antibodies (Santa Cruz Biotechnology, Inc.) and dilutions for immunoblotting of ovine amnion membrane protein

Target protein	Primary antibody product no.	Isotype	Dilution	Secondary antibody product no.	Isotype	Dilution
AQP1	sc‐9878 (L‐19)	Goat polyclonal IgG	1:250	sc‐2304	Donkey anti‐goat HRP polyclonal IgG	1:40,000
AQP3	sc‐9885 (C‐18)	Goat polyclonal IgG	1:250	sc‐2304	Donkey anti‐goat HRP polyclonal IgG	1:40,000
AQP8	sc‐28624 (H‐85)	Rabbit polyclonal IgG	1:20,000	sc‐2054	Goat anti‐rabbit HRP polyclonal IgG	1:40,000
AQP9	sc‐74409 (G‐3)	Mouse monoclonal IgG2a, broad spectrum	1:500	sc‐2970	Goat anti‐mouse HRP monoclonal IgG2a	1:40,000
AQP11	sc‐138132 (N‐14)	Goat polyclonal IgG	1:500	sc‐2304	Donkey anti‐goat HRP polyclonal IgG	1:40,000
Β‐actin	sc‐69879 (AC‐15)	Mouse monoclonal IgG1, broad spectrum	1:5x10^5^	sc‐2969	Goat anti‐mouse HRP monoclonal IgG1	1:40,000

### Statistical analysis

Data are presented as mean ± SE and were compared statistically with one‐factor analysis of variance (ANOVAs). Post hoc testing utilized Fisher's least significant difference if the null hypothesis was rejected with the ANOVAs. Relationships between IMA rate, AFV, and AQP mRNA and protein levels were determined with least squares bivariate and multivariate regression analyses. The coefficient of determination (*r*²) was expressed as r squared times 100%. For analysis of the AQP mRNA levels, base 10 logarithmic transformation was used to normalize variances prior to statistical testing and the data were plotted on a logarithmic scale. For mRNA levels, analysis of the log values was equivalent to the analysis of direct values for ΔC_T_ and ΔΔC_T_ as identical *P* values were obtained. *P* < 0.05 was considered significant.

## Results

**Figure 1:**
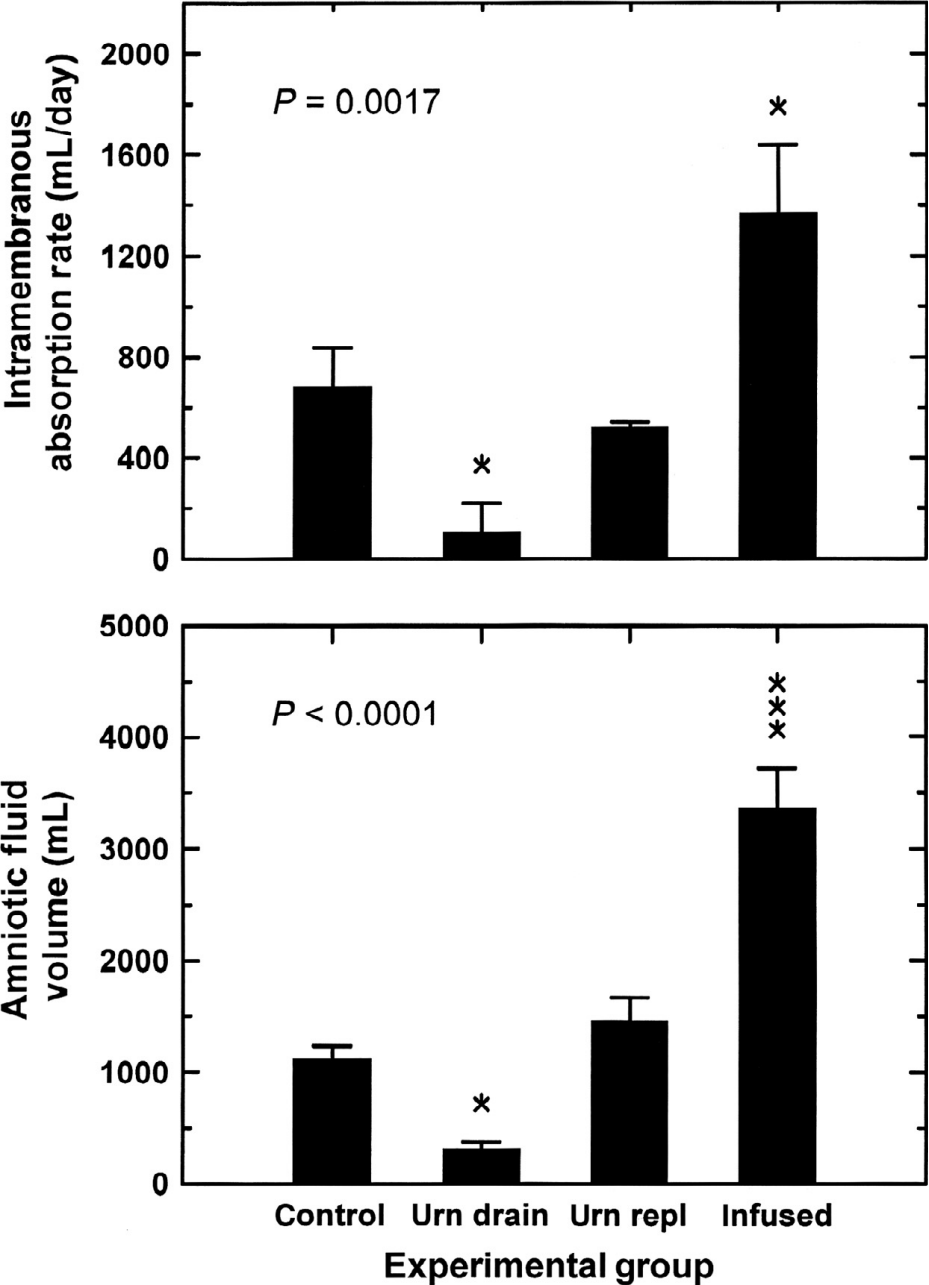
Intramembranous absorption rate and amniotic fluid volume as a function of experimental conditions: urn drain, fetal urine drainage; urn repl, urine drainage and isovolumic replacement with lactated Ringer's solution; infused, intraamniotic infusion of lactated Ringer's solution. Intramembranous absorption rate was an average over the 2‐day experimental period, amniotic fluid volume was the volume at the end of experimental period. *P* values are from one‐factor analysis of variance (ANOVA). Post hoc comparisons: *P < 0.05; ***P < 0.001 compared to control conditions.

During control conditions, AFV was 1,110 ± 125 mL, while IMA rate averaged 667 ± 159 mL/day (*n* = 4). These values were altered under experimental conditions. Urine drainage reduced and intraamniotic fluid infusion increased IMA rate (*P* < 0.01) as well as AFV (*P* < 0.0001) when compared to control conditions (Fig. 1). With urine replacement, the reduction in mean IMA rate and increase in mean AFV were not significant when compared to control.

**Figure 2:**
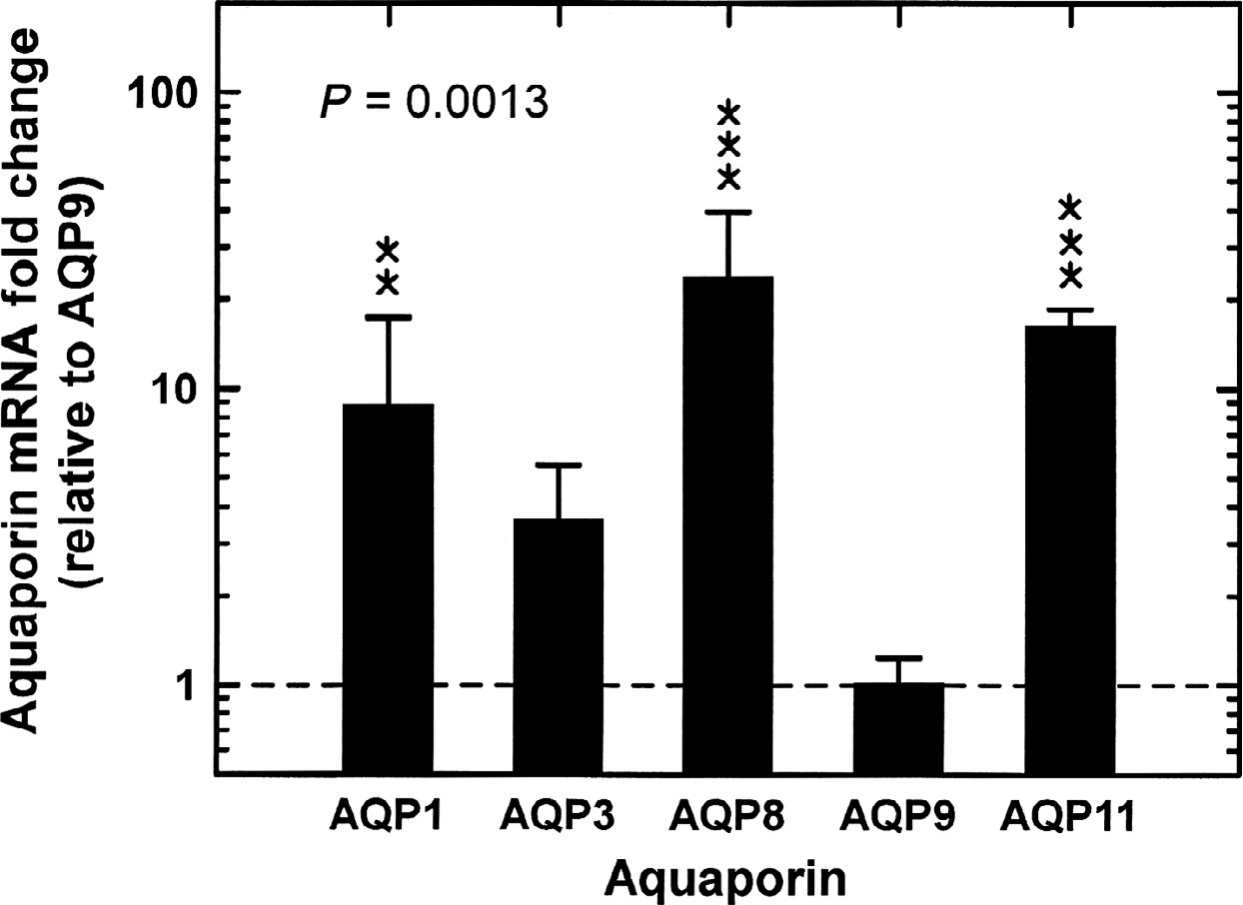
Relative aquaporin mRNA levels (expressed as fold change from AQP9) in ovine amnion under control conditions. Values were calculated using the ΔΔC_T_ method and relative to control AQP9 mRNA levels (horizontal dashed line). Data were analyzed with a one‐factor ANOVA. Post hoc comparisons for each aquaporin: **P < 0.01; ***P < 0.001 compared to AQP9.

**Figure 3:**
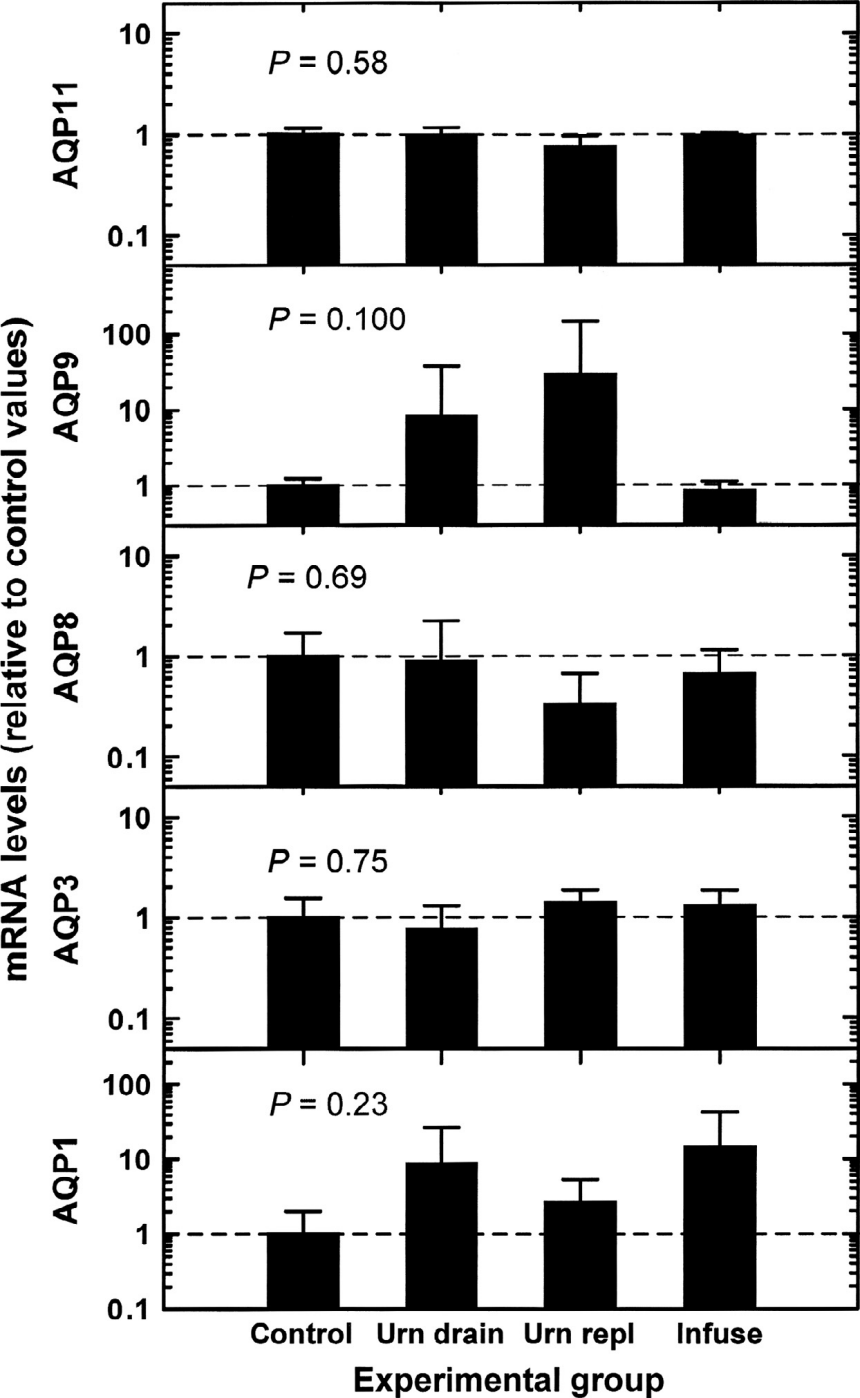
AQP mRNA levels during experimental conditions relative to control mRNA level for each AQP. Experimental conditions were: urn drain, fetal urine drainage; urn repl, urine drainage and isovolumic replacement with lactated Ringer's solution; infuse, intraamniotic infusion of lactated Ringer's solution. Data were normalized to mean value for each AQP mRNA during control conditions (horizontal dashed lines). *P* values are from one‐factor ANOVA.

In the amnion, under control conditions, there were large differences (*P* = 0.0013) in AQP mRNA abundance among the five AQPs (Fig. 2). AQP9 mRNA levels were least expressed, while AQP8 mRNA levels were highest averaging 23 times that of AQP9. AQP1, AQP3, and AQP11 mRNA levels were intermediate between these levels. This differential pattern of mRNA levels for the five AQPs was also detected in the remaining three experimental groups. There were no significant differences in mRNA levels for each AQP between control and individual experimental groups or among groups (Fig. 3).

Although mean IMA rate ranged from a low of 100 ± 120 mL/day during urine drainage to a high of 1370 ± 270 mL/day during intraamniotic infusion, the mRNA levels of AQP1 (*r* = 0.22, *P* = 0.42), AQP3 (*r* = −0.08, *P* = 0.77), AQP8 (*r* = −0.11, *P* = 0.68), AQP9 (*r* = −0.32, *P* = 0.25), and AQP11 (*r* = −0.11, *P* = 0.97) were not correlated with IMA rate. With multivariate regression analysis of the five AQPs in all four groups combined (*n* = 16), there were no significant relationships among IMA rates and AQP mRNA levels.

**Figure 4:**
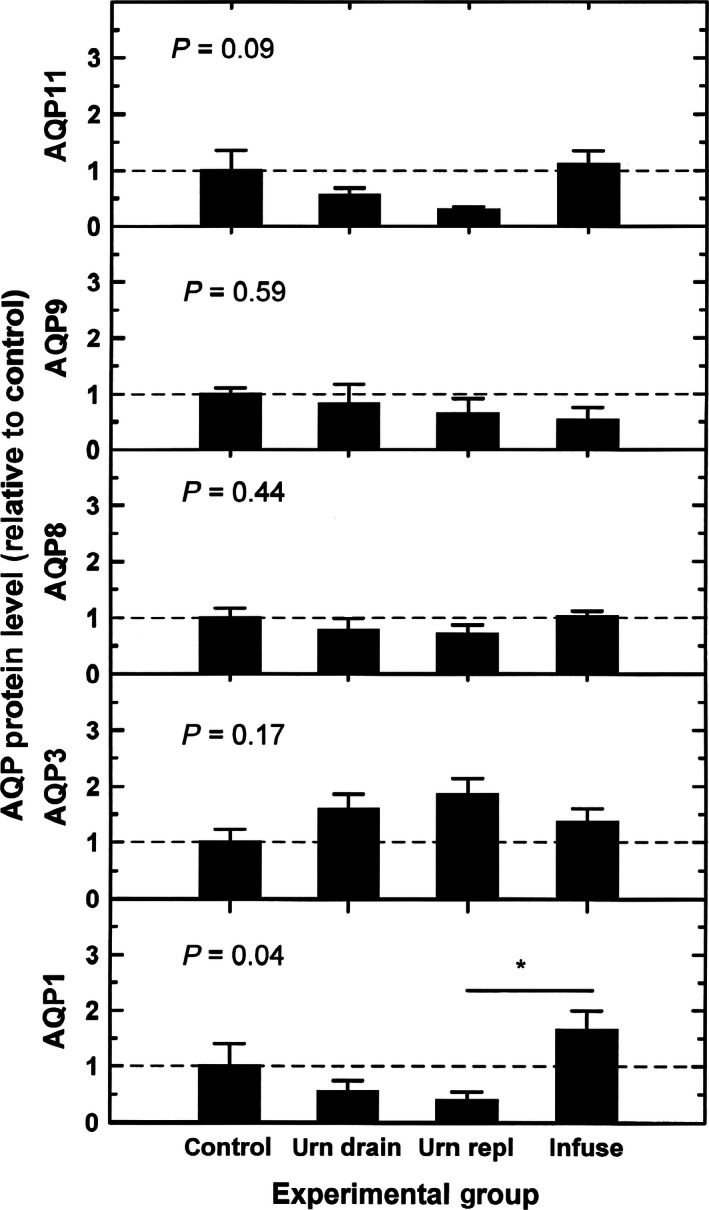
AQP protein levels during experimental conditions relative to control protein levels for each AQP. Experimental conditions were: urn drain, fetal urine drainage; urn repl, urine drainage and isovolumic replacement with lactated Ringer's solution; infuse, intraamniotic infusion of lactated Ringer's solution. Data were normalized to mean value for each AQP protein during control conditions (horizontal dashed lines). *P* values are from one‐factor ANOVA. *P < 0.05.

**Figure 5:**
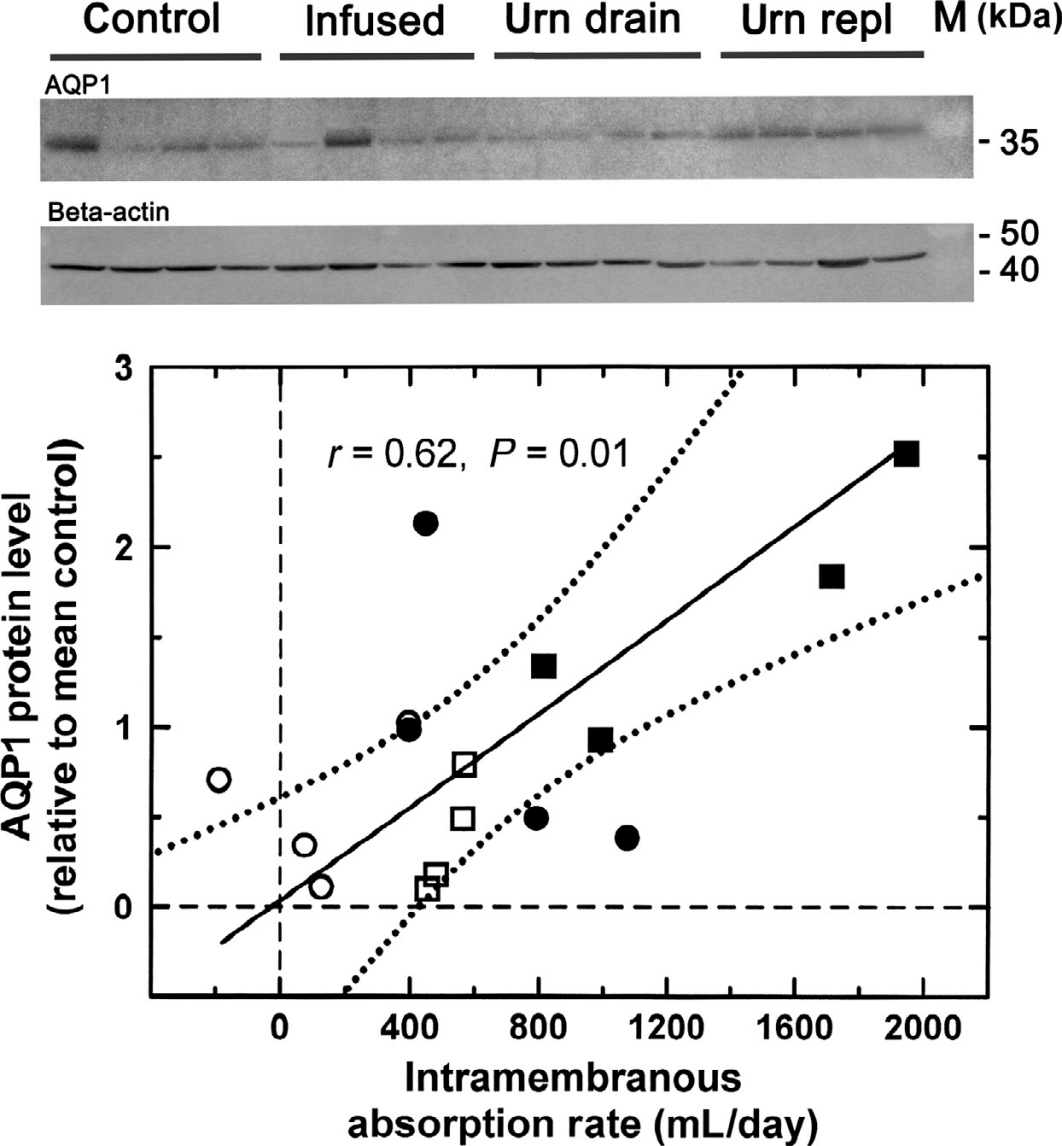
AQP1 protein expression in ovine amnion. Upper panel: representative western gel of AQP1 protein in the four experimental groups. Lower panel: bivariate regression relationship between AQP1 protein levels of four experimental groups and intramembranous absorption rates. Individual AQP1 protein values were normalized to the mean AQP1 value in the control group. Filled circle, control conditions; open circle, urine drainage; open square, urine drainage and isovolumic replacement with lactated Ringer's solution; filled square, intraamniotic fluid infusion of lactated Ringer's solution.

The protein levels of each of the five AQPs in the three experimental groups were not significantly different from that in the control group (Fig. 4). For AQP1 protein, a significant difference was noted between urine replacement group and intraamniotic infusion group. Using bivariate regression on the combined data from all four groups, there was a positive relationship between AQP1 protein level and IMA rate (*r* ^2^ = 38%, *P* = 0.01, Fig. 5). However, the protein levels of AQP3 (*r* = −0.31, *P* = 0.24), AQP8 (*r* = 0.20, *P* = 0.45), AQP9 (*r* = −0.30, *P* = 0.26), and AQP11 (*r* = 0.32, *P* = 0.23) were not statistically related to IMA rate. With multivariate regression, IMA rate was positively correlated only with AQP1 protein levels, but not with the other AQP proteins. AFV was not significantly correlated with any of the five AQP mRNA or protein levels in the amnion.

With the combined data for the five AQPs from all four groups (*n* = 16 for each AQP), the relationships between mRNA and protein levels for individual AQP in the amnion were analyzed. For AQP1, mRNA levels were not significantly correlated with protein levels (*P* = 0.62). Similarly, there were no significant correlations between mRNA and protein levels for AQP3 (*P* = 0.67), AQP8 (*P* = 0.35), AQP9 (*P* = 0.43), or AQP11 (*P* = 0.10).

## Discussion

A primary objective of this study in ovine fetuses was to determine whether there are correlations between AQP mRNA and/or protein levels and IMA rate as would be consistent with the concept that AQPs regulate IMA and ultimately AFV. If the AQPs participate in the dynamic regulation of AFV, AQP protein levels within the amnion would be expected to positively correlate with the rate at which fluid is transported across the amnion over a wide range of IMA rates. A unique finding in this study is that AQP1 protein levels and IMA rate are indeed positively correlated while the other AQP proteins are not. The positive relationship suggests that AQP1 may act to facilitate water transfer across the amnion to regulate IMA rate, and thus modulate AFV. These results support the hypothesis that AQP1 may participate in the dynamic regulation of AFV as previously speculated (Liu and Wintour 2005; Beall et al. 2007a,b; Liu et al. 2008). Although the extent to which AQP1 contributes to overall AFV regulation has yet to be determined, a role for AQP1 in AFV regulation is consistent with the reported phenotype of mice carrying homozygous deletions of the AQP1 gene. In this knockout mouse model, AFV increased by 27% when compared to wild‐type animals (Mann et al. 2005). However, whether this increase was due to changes in IMA rate, placental function, fetal swallowing, renal excretion, or other factors is unknown. Furthermore, it is unclear whether the observed changes were the result of a global AQP1 gene deletion and the associated changes in placental degeneration in addition to reductions in fetal weight (Zheng et al. 2014), both of which may alter fetal fluid status and hence AFV.

This study in pregnant sheep supports a role of AQP1 in the regulation of IMA rate under conditions of altered AFV. However, it should be emphasized that the functional role of AQP1, AQP3, AQP8, AQP9, and AQP11 in amniotic fluid transport under normal conditions has not been established. Our early studies in ovine fetuses found that water administered into amniotic fluid rapidly crosses the intramembranous pathway (Gilbert and Brace, 1989, 1990). This demonstrates that water flux through AQP channels is clearly important. Recent studies of intramembranous water and solute fluxes suggest that, under basal conditions, intramembranous water flow presumably facilitated by AQPs is a passive component that constitutes 15–20% of the overall volume transport across the amnion, whereas the unidirectional vesicular IMA is large and is the primary component of the amniotic fluid transport (Anderson et al. 2013; Brace et al. 2014b). These findings together with current data suggest that passive water movement through AQP1 water channels in the ovine amnion may be important in dynamically regulating IMA rate primarily when AFV is altered.

A second aspect of this study was to explore whether the five AQPs (AQP1, AQP3, AQP8, AQP9, and AQP11) found in human amnion (Prat et al. 2012; Bednar et al. 2015) are similarly expressed in the ovine amnion. In our previous studies of AQP gene expressions in the human amnion, we found differences in expression pattern between placental and reflected amnion. Furthermore, there were differential expression profiles for the five AQPs within each region of the amnion (Bednar et al. 2015). Our present results confirmed that both mRNA and protein of the five AQPs that are present in human amnion are also expressed in the ovine amnion. A new observation is that the expression pattern of the five AQPs in the amnion is distinctly different between humans and sheep. In sheep, AQP8 mRNA levels were the highest among the five AQPs while AQP9 mRNA levels were least expressed, whereas in humans, AQP8 was least expressed and AQP9 mRNA was expressed at high levels (Bednar et al. 2015). This implies that individual AQPs in the amnion may provide species‐specific contributions to water and solute transport across the amnion.

Our studies did not detect any changes in AQP mRNA levels under conditions of altered IMA rates or AFVs. In previous reports of abnormal AFV conditions in human pregnancies, AQP1, AQP3, AQP8, and AQP9 mRNA levels were found to be reduced in subjects with oligohydramnios (Zhu et al. 2009; Jiang et al. 2012) while AQP1, AQP8, and AQP9 mRNA levels were elevated in conditions of polyhydramnios (Mann et al. 2006; Zhu et al. 2010). In those studies, the AQP protein levels were not reported, thus it is not possible to ascertain the functional significance of the findings. Those observations of AQP mRNAs in humans were not supported by the gene expression data in the present pregnant sheep studies under conditions of comparable increases or decreases in AFVs. The difference in findings may be related to species differences but possibly to the duration of altered AFV. Our studies determined changes after 2 days of AFV modifications while in the human studies, the period of oligohydramnios or polyhydramnios were not stated. In addition, whether the conditions of abnormal AFVs were complicated by comorbidities in the human studies is not known.

One potential concern with the present conclusions is the limited sample size, with four subjects in each of the four groups for a total of 16 animals. This can be addressed by recalculating the statistical comparisons assuming the means, variances, and correlation coefficients are unchanged when the sample size doubled (*n* = 8 for each of four groups). The two primary questions that we addressed in this study were (1) are there differences in the relative expression among the five AQPs within the amnion and (2) do AQP mRNA and protein levels correlate with IMA rate when IMA rate is experimentally altered? With respect to the first question, the recalculation indicated that in the control group, the highly significant difference in AQP mRNA levels (*P* < 0.0001) remained with the added result that the difference in mRNA levels between AQP3 and AQP9 reached statistical significance. As for the second question, the recalculation similarly showed that AQP1 protein remained positively correlated with IMA rate (*P* = 0.00015), but the remaining four AQP proteins were not. Furthermore, AQP mRNA levels remained uncorrelated with IMA rate. These analyses are consistent with the primary conclusions of this study. Thus, the sample sizes of *n* = 4 for each of four groups with *n* = 16 combined were adequate to address our primary questions.

In summary, we documented the gene expression of five AQPs in sheep amnion and determined their differential expression profiles under conditions of normal and abnormal AFVs. Our studies did not demonstrate a correlation between relative mRNA levels for individual AQPs and IMA rate or AFV. The major finding in this study was a significant positive relationship between IMA rate and AQP1 protein levels in the amnion under combined conditions of normal and abnormal IMA rates. These results support the concept that AQP1 may be an important regulator of amniotic fluid transport across the amnion when IMA rate is altered. As such, AQP1 may play a significant role in the maintenance of AFV within the normal range.

## Conflict of Interest

There are no conflicts of interest for any of the authors.
